# Parkinson’s Disease Detection from Resting-State EEG Signals Using Common Spatial Pattern, Entropy, and Machine Learning Techniques

**DOI:** 10.3390/diagnostics12051033

**Published:** 2022-04-20

**Authors:** Majid Aljalal, Saeed A. Aldosari, Khalil AlSharabi, Akram M. Abdurraqeeb, Fahd A. Alturki

**Affiliations:** Department of Electrical Engineering, King Saud University, Riyadh 11421, Saudi Arabia; dosari@ksu.edu.sa (S.A.A.); kabdulghani@ksu.edu.sa (K.A.); amohammed6@ksu.edu.sa (A.M.A.); falturki@ksu.edu.sa (F.A.A.)

**Keywords:** common spatial pattern, discriminant analysis, electroencephalogram, entropy, k-nearest neighbor, machine learning, Parkinson’s detection, random forest, support vector machines

## Abstract

Parkinson’s disease (PD) is a very common brain abnormality that affects people all over the world. Early detection of such abnormality is critical in clinical diagnosis in order to prevent disease progression. Electroencephalography (EEG) is one of the most important PD diagnostic tools since this disease is linked to the brain. In this study, novel efficient common spatial pattern-based approaches for detecting Parkinson’s disease in two cases, off–medication and on–medication, are proposed. First, the EEG signals are preprocessed to remove major artifacts before spatial filtering using a common spatial pattern. Several features are extracted from spatially filtered signals using different metrics, namely, variance, band power, energy, and several types of entropy. Machine learning techniques, namely, random forest, linear/quadratic discriminant analysis, support vector machine, and k-nearest neighbor, are investigated to classify the extracted features. The impacts of frequency bands, segment length, and reduction number on the results are also investigated in this work. The proposed methods are tested using two EEG datasets: the SanDiego dataset (31 participants, 93 min) and the UNM dataset (54 participants, 54 min). The results show that the proposed methods, particularly the combination of common spatial patterns and log energy entropy, provide competitive results when compared to methods in the literature. The achieved results in terms of classification accuracy, sensitivity, and specificity in the case of off-medication PD detection are around 99%. In the case of on-medication PD, the results range from 95% to 98%. The results also reveal that features extracted from the alpha and beta bands have the highest classification accuracy.

## 1. Introduction

With age, the number of connections between brain cells reduces and the neurons shrink. Nerve cells, unlike muscle, skin, and bone cells, cannot regenerate themselves. Neurons die or become damaged as people age [1]. Parkinson’s disease (PD) is a neurodegenerative disease in which neurons in the substantia nigra of the brain become damaged. These neurons are in charge of producing a substance known as dopamine. Dopamine is a chemical that acts as a messenger between neurons in the brain. It assists the brain in sending messages to various regions of the body in order for it to work properly, particularly when it comes to body movements and speech delivery. PD symptoms appear when a high number of dopaminergic neurons are destroyed or the quantity of dopamine in the brain is abnormal [2]. According to the World Health Organization, around 10 million individuals have been affected as a result of this disease [3,4]. PD becomes more common as people get older, with people in their fifties and older being the most affected. Approximately 4% of people with PD are diagnosed before they reach the age of 50, and males are 1.5 times more likely than women to have the disease [5,6]. Early symptoms may be minor and difficult to notice, but as time passes, the signs and symptoms will become more noticeable. Dyskinesia, fainting, exhaustion, tremor, rigidity, dystonia, hypomimia, constipation, loss of taste or smell, and weight loss are some of the motor and non-motor symptoms of PD [6]. Because PD is currently incurable, it is essential to detect it early so that patients can take the necessary preventative measures to manage it and carry out their regular activities properly [6,7].

Parkinson’s disease manifests itself in a variety of ways for those who have it. Its symptoms may not always appear in the same order. The most popular scales used to score the disease and assess the phases of Parkinson’s disease are the Hoehn and Yahr (HY) rating scale and the Unified Parkinson’s Disease Rating Scale (UPDRS) [8]. The HY scale classifies Parkinson’s disease into five stages, ranging from no symptoms to the most dangerous stage. Similarly, UPDRS categorizes PD into five categories, starting from zero, which denotes normal, and progressing to the fourth, which denotes significant difficulties [6,9].

Even while the final diagnosis is always subject to the neurologist’s opinion and review, any tool that helps them contrast their diagnosis is always welcome. As a result, there is a growing demand for automated procedures that can aid in improving the accuracy of PD diagnosis. Several approaches have been presented in this regard, with the majority of them using voice signals [10,11,12], gait signals [13,14], handwriting signals [15,16], MRI [17], and only a few employing electroencephalography (EEG). EEG is considered to be one of the most important PD diagnostics tools. EEG technology can be used to capture cerebral information in a real-world context because it is reasonably inexpensive and portable. In addition, EEG records brain activity faster and for a longer amount of time than other technologies. As a result, EEG analysis, along with machine learning techniques, has already been employed in the detection of several neurological conditions, including epilepsy, autism spectrum disorder, Alzheimer’s disease, schizophrenia, and major depressive disorder [18,19,20,21,22,23]. However, the use of EEG to study PD has not been fully investigated.

During Timed Up and Go Task studies, Ly et al. [24] reported a classification approach based on EEG data for detecting turning freeze (TF) occurrences in six PD patients. For feature extraction, time-frequency Stockwell transform techniques were applied. Independent component analysis with entropy bound minimization was used to separate the EEG sources. Bayesian Neural Networks were used to extract and classify the different frequency-based features of selected independent components of EEG, which provided 86.2% accuracy for TF detection. Another study [25] discussed various ways of identifying gait initiation failure (GIF), which is a sort of freezing of gait (FOG). In five PD patients with FOG, wavelet transform was employed for feature extraction, after which GIF events were classified using a support vector machine. With an accuracy of 86.3%, these approaches were able to correctly identify GIF episodes. The study [26] described deep learning models for diagnosis derived from resting EEG data collected from patients with Rapid Eye Movement Behavior Disorder. The authors utilized RNN-LSTM and five-layer CNN to obtain classification accuracy of 81% and 79%, respectively.

Chaturvedi et al. [27] aimed to find out which quantitative EEG (QEEG) parameters could best distinguish patients with PD from healthy individuals. For this purpose, 256-channel EEG signals from 50 PD and 41 healthy controls (HC) were processed using regression and machine learning methods. Betrouni et al. [28] studied the optimal QEEG characteristics for detecting different levels of cognitive impairment in Parkinson’s disease. Spectral power analysis was performed on the EEG recordings, and characterization models were built and trained using support vector machines and k-nearest neighbors. The total classification accuracies for the support vector machines and k-nearest methods were 84% and 88%, respectively.

The studies discussed so far are either studies that focus on detecting Parkinson’s disease by asking participants to perform mental or muscular tasks [24,25,26] or studies that focus on finding out the best EEG parameters/characteristics for PD detection [27,28]. The following studies are dedicated to the detection of PD patients from HC using resting-state EEG recordings. Starting with Yuvaraj et al. [29], they employed a higher-order spectra (HOS) feature extractor to develop an automated diagnosis of Parkinson’s disease. The bispectrum features were retrieved and their relevance was assessed. Decision tree, KNN, Fuzzy-KNN, NB, Probabilistic neural network, and SVM are some of the classifiers that produce accuracy ranging from 90.6% to 99.6%. The use of resting-state EEG signals to detect Parkinson’s disease has also been investigated in [30]. In that study, a thirteen-layer convolutional neural network (CNN) was proposed, achieving a classification accuracy of 88.25%. For the categorization of on–medication PD (on–PD) vs. off–medication PD (off–PD) patients, Shah S. A. A. et al. [31] developed a deep neural network architecture termed the dynamical system generated hybrid network (DGHNet). They reported that this network has a classification accuracy of 99.2%. Linear predictive coding (LPC) was proposed by Md. Fahim Anjum et al. [32] to distinguish spectral EEG markers of Parkinson’s disease. The power spectral density (PSD) of EEG data was computed, then LPC was employed for feature extraction. The resulting LPC vectors are used to identify two separate hyperplanes, which are then used to distinguish PD patients from healthy controls with an accuracy of 85.3%. Lee S. et al. [33] proposed a convolutional neural network (CNN) and a recurrent neural network (RNN) with gated recurrent units (GRUs) for identifying resting-state EEG obtained from people with PD and HC in a recently published study. Their proposed approach has a 99.2% classification accuracy. The wavelet transform was proposed by Smith K. K. et al. [34] to decompose EEG signals into several subbands. Statistical measurements were used to extract five features from these subbands, which were then categorized using several machine learning techniques. The classification of off-medication PD vs. HC and on-medication PD vs. HC using the least square support vector machine yielded an accuracy of 96.13% and 97.65%, respectively.

Although the detection of PD patients in the resting state is more comfortable for elderly people, there are a limited number of studies [29,30,31,32,33,34] that were dedicated to that. For comparison purposes, Table 1 summarizes these studies with their proposed feature extraction and classification methods and corresponding results. However, it should be noted that at least three different datasets were used in these studies: the Malaysian dataset, public UNM dataset, and public SanDiego dataset, making the comparison difficult. Looking first at the classification accuracy results for these studies in the table, it can be noted that the results of the studies are varying and some of them have high classification accuracy. By looking again at the table, some of these studies [30,31,33] used deep learning techniques, which could provide high classification accuracy, but these techniques require a long training period and a big dataset as well, which makes them unsuitable for implementation in reality. From Table 1, it can also be seen that some studies, such as [32], suggested the use of uncomplicated methods, but on the other hand, the simplicity had a negative impact on the accuracy of classification.

The aim of the present study is to address these gaps found in previous studies by presenting uncomplicated feature extraction and classification methods while maintaining high classification accuracy and validating them using two public datasets (UNM and SanDiego datasets). It is worth mentioning that the classification accuracy is influenced not just by the classifier utilized but also by the signal’s preprocessing and the method of extracting features. In our recent study [35], a CSP-based diagnostic method for identifying epilepsy and ASDs was developed, and the results were promising. These results motivated us to investigate if the CSP approach may yield good biomarkers of PD patients’ resting-state EEGs, allowing them to be distinguished from those of healthy people.

Accordingly, in the present study, novel, simple, and effective CSP-based methods are proposed for the detection of PD in two conditions, namely, off–medication PD vs. HC and on–medication PD vs. HC. To the best of our knowledge, we are the first group to present CSP-based methods for the detection of PD. Unlike traditional CSP-variance, CSP is combined with various methods to improve classification accuracy, including energy and band power (BP). In addition, unlike [35], CSP is also combined with log energy entropy, norm entropy, sure entropy, and Shannon entropy, to provide good biomarkers for PD EEGs. Several linear/nonlinear classifiers are applied to classify the resulting PD features from normal ones. The effects of the frequency band, reduction number, and segment length on classification accuracy are also being investigated.

The rest of this paper is laid out as follows. Section 2 describes the used EEG data and the following EEG signal-processing methods: preprocessing, feature extraction, and classification techniques. The rest of this paper is laid out as follows. Section 3 contains the results as well as a discussion. In Section 4, the conclusion is presented, as well as potential future work options.

## 2. Methods

**Figure 1:**
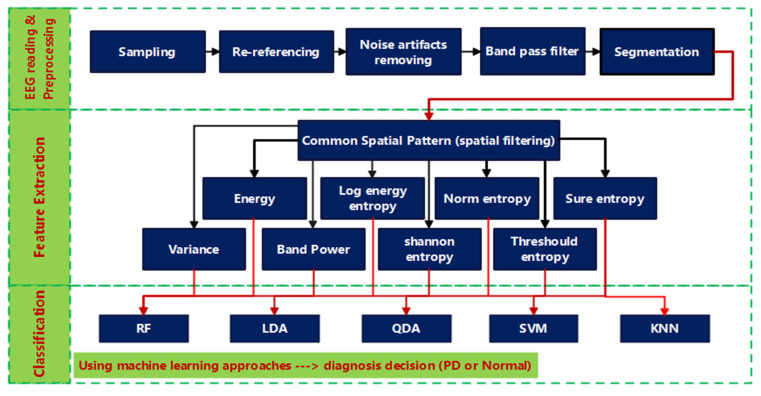
Block diagram of the proposed PD CSP-based classification method.

The proposed methods for processing EEG signals are described in this section, which includes data description, preprocessing, feature extraction, and classification methods. Figure 1 provides a high-level overview of the different stages through which EEGs from Parkinson’s patients and healthy people are analyzed and then classified. The raw EEG signals are read first, then preprocessed to remove artifacts before being band-pass filtered to find the frequency band of interest. The filtered EEG signals are split into non-overlapping segments with an equal time duration. Each segment is spatially filtered using CSP, after which the PD/HC features are extracted using a variety of metrics including variance, band power, energy, log energy entropy, norm entropy, threshold entropy, sure entropy, and Shannon entropy. Finally, to distinguish off/on PD features from HC ones, various classifiers such as random forest (RF), linear discriminant analysis (LDA), quadratic discriminant analysis (QDA), support vector machine (SVM), and k-nearest neighbors (KNN) are used (HC). The subsections that follow go over each stage of the block diagram in further detail.

### 2.1. Data Description and Pre-Processing

In this study, the proposed methods are tested using two public EEG datasets. The first dataset is from the University of San Diego, California [36]. This dataset is referred to as the “SanDiego dataset.” The subjects of this dataset were asked to sit comfortably and relax their eyes by fixating on a cross on a screen during data collection. There are two groups in the dataset. The EEGs of 16 healthy subjects with a mean age of 63.5 ± 9.6 standard deviation years, 9 females and 7 males, make up the first group. The EEGs of 15 PD patients, 8 females and 7 males, with a mean age of 63.2 ± 8.2 standard deviation years, make up the second group. As determined by the Mini-Mental State Exam and the North American Adult Reading Test, the right-handedness, gender, age, and cognition of the PD patients were quite similar to those of the HC. All of the patients had mild to severe Parkinson’s disease (Hoehn and Yahr scale II and III), with an average disease duration of 4.5 to 3.5 years. To get information on EEG on and off medication, data from PD patients was obtained on two separate days. For the on-medication session, the participants brought their usual medication regimen with them to the recording session. The patients had been taking their medications for about 12 h when they agreed to participate in the off-medication session. The healthy subjects only volunteered once. Using a 32-channel Biosemi Active Two EEG system, EEG signals were recorded for at least 3 min at a sampling frequency of 512 Hz. In addition to the 32 EEG channels, each recording has eight EXG channels. The preprocessing was conducted in Matlab using EEGLAB by removing the mean of each channel and re-referencing all of the data to the common average (excluding excessively noisy electrodes). To reduce low-frequency drift, high pass filtering at 0.5 Hz was applied. Eye blinks and movements, muscle activity, electrical noise, and other sorts of noise were manually examined and removed. This dataset’s specifics, including signal capture and preprocessing, are detailed in [37].

The second set of data comes from a study conducted by the University of New Mexico (UNM; Albuquerque, NM, USA). For simplicity, in the present study, this dataset is referred to as the UNM dataset. This dataset contains the EEGs of 27 Parkinson’s disease patients and 27 healthy subjects of equal gender (17 females and 10 males). The mean age plus standard deviation for the PD group is 69.52 ± 8.56 years, while the mean age plus standard deviation for the HC group is 69.52 ± 9.27 years. In terms of age and sex, control subjects and PD patients were demographically matched, and no variations in education or premorbid IQ were discovered. The PD group visited the lab twice, seven days apart, the first time while on medication and the second time after a 15-h overnight withdrawal from their individual dopaminergic pharmacological prescriptions. As a result, the data set includes information from 27 Parkinson’s disease patients who were on and off therapy. Data were collected for two minutes for each patient and control group; first, they were instructed to keep their eyes closed for one minute, and then they were asked to record for another minute with their eyes open. For a total of 68 channels, sintered Ag/AgCl electrodes were used for 64 EEG channels, 65 VEOG channels, and 65–67 XYZ accelerometer channels on hand (variable L or R). The sampling rate was 500 samples per second. The Brain Vision data collection system was used with an online CPz reference and an AFz terminal grounded. The paper [38] goes into greater detail about how the data was gathered. Table 2 provides a summary of both the SanDiego and the UNM datasets.

**Figure 2:**
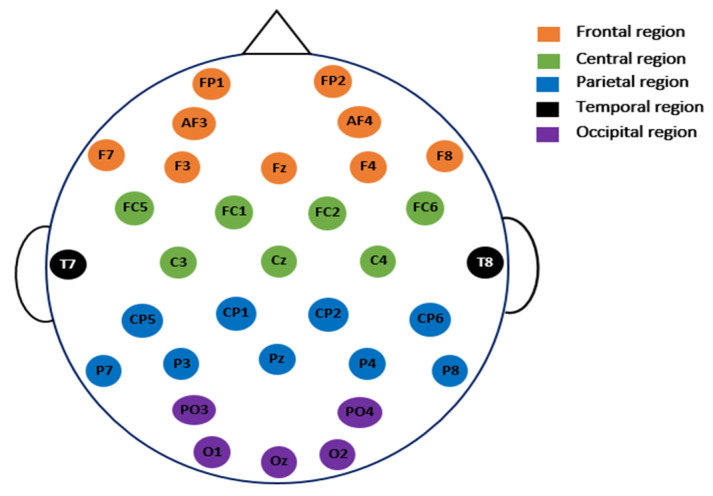
The placement of the electrodes for the 32 EEG channels used in SanDiego dataset.

**Figure 3:**
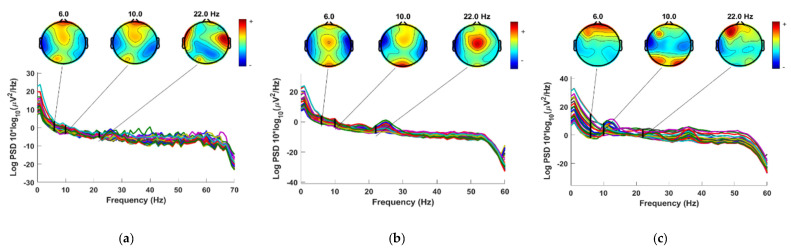
Power spectral density and electrode map for (**a**) Off−PD EEG (**b**) On−PD EEG (**c**) HC EEG.

In this study, certain superfluous channels were eliminated from the SanDiego and UNM datasets. The 8 EXG channels (non-EEG channels) were removed from the SanDiego dataset. As a result, each individual has only 32 EEG channels, as shown in Figure 2. The VEOG channel and the XYZ accelerometer have also been removed from the UNM dataset. Figure 3 depicts electrode maps and EEG power spectral density (on a logarithmic scale) for off–PD, on–PD, and HC EEGs. The electrode map is shown for three distinct arbitrary frequencies: 6, 10, and 22 Hz. In general, the power density of the low-frequency spectrum is higher than that of the high-frequency spectrum. Different power spectral density patterns can be seen when comparing the three maps.

For further preprocessing, the EEG signals are divided into M segments with a size of (ch×N), where ch denotes the number of channels and N specifies the number of EEG samples per channel in a given time interval T. The segmented signals are then filtered with a fifth-order band-pass Butterworth filter to remove the interference and noise caused by the electrodes and magnetic fields. The choice of the segmentation time interval T and the frequency band of the filter will be investigated later in this paper.

### 2.2. Common Spatial Pattern

For discriminating between the off/on PD class and HC class, the CSP algorithm is employed as a spatial filter that leads to peak variances [39]. For simplicity, in what follows, these two classes will be denoted by PD and HC, respectively. A set of CSP filters make up the projection matrix $W_{CSP}$, which is computed only once using the entire training dataset. This is done by first calculating the normalized spatial covariance for both classes as follows

(1) $$C_{PD}=\frac{E_{PD}E_{PD}{}^{\prime }}{trace\left(E_{PD}E_{PD}{}^{\prime }\right)}\;\;\;and\;\;\;\;C_{HC}=\frac{E_{HC}E_{HC}{}^{\prime }}{trace\left(E_{HC}E_{HC}{}^{\prime }\right)}\;$$

where $E_{PD}$ and $E_{HC}$ denote the EEG segments under two conditions (PD and HC) of size ch×N, where ch denotes the number of channels and N denotes the number of samples per channel in each segment. $E^{\prime }$ is the transpose of E, and $trace\left(EE{}^{\prime }\right)$ is the sum of the diagonal elements of $EE^{\prime }$. Then, the averaged normalized covariances $\bar{C_{PD}}$ and $\bar{C_{HC}}$ are calculated by averaging all of the segments of each class. The overall composite spatial covariance is given by

(2) $$C_{C}=\bar{C_{PD}}+\bar{C_{HC}}\;$$

This covariance matrix is factorized into eigenvalues and eigenvectors as follows

(3) $$C_{C}=U_{C}\lambda_{C}{U^{\prime }}_{C}$$

where $U_{C}$ is the matrix of the eigenvectors and $\lambda_{C}$ is the diagonal matrix of the eigenvalues arranged in descending order. Subsequently, the whitening transformation P is obtained by computing

(4) $$P=\sqrt{\lambda_{C}^{-1}}\;U^{\prime }{}_{C}$$

This is used to transform the covariance matrices of the two classes into

(5) $$S_{PD}=P\;\bar{C_{PD}}\;P^{\prime }\;\;\;\;and\;\;\;\;\;S_{HC}=P\;\bar{C_{HC}}\;P^{\prime }$$

The sum of the eigenvalues of $S_{PD}$ and $S_{HC}$ should be an identity matrix, and $S_{PD}$ and $S_{HC}$ should have the same eigenvectors, i.e.,

(6) $$S_{PD}=B\;\lambda_{PD}B^{\prime }$$

(7) $$S_{HC}=B\;\lambda_{HC}B^{\prime }$$

(8) $$\lambda_{PD}+\lambda_{HC}=I$$

where *B* is any orthonormal matrix that satisfies

(9) $$B^{\prime }\left(S_{PD}+S_{HC}\right)B=I$$

The eigenvector corresponding to the largest eigenvalue for $S_{PD}$ have the smallest eigenvalues for $S_{HC}$, and vice versa. This demonstrates that the maximization of the eigenvalues of one class at a specific point corresponds to the minimization of the eigenvalues of the other class at the same point. Thus, the covariance between the two classes is successfully maximized. The projection matrix $W_{CSP}$ is defined by

(10) $$W_{CSP}=P^{\prime }B=\left[\;w_{1}\;w_{2}\;\ldots \;w_{ch-1}\;w_{ch}\right]\in R^{ch\times ch}$$

which is composed of a set of CSP filters. The first CSP filter $w_{1}$ corresponds to the maximum variance of PD class while the last CSP filter $w_{ch}$ provides the maximum variance of HC class. For dimensionality reduction, only the first and last m filters will be used, such that $W_{CSP}$ is redefined as follows

(11) $$W_{CSP}=\left[\;w_{1}\;w_{2}\;\ldots \;w_{m}\;\;w_{ch-m+1}\;w_{ch-m+2}\;\ldots \;w_{ch}\right]\in R^{d\times ch}$$

where d=2m is the reduction number. The reduction number is the number by which the channels should be reduced. The process of feature extraction starts by filtering each EEG segment using *W_CSP_* to obtain the filtered segment S is given by

(12) $$S=W_{CSP}^{2m\times ch}\;e{\left(t\right)}^{ch\times N}={\left[s_{1}\left(n\right)\;s_{2}\left(n\right)\;\ldots \;s_{d}\left(n\right)\right]}^{\prime }\;\in R^{2m\times N}$$

### 2.3. Feature Extraction (FE)

In conventional CSP, the variance measurement is used to calculate the feature vectors $f={\left(f_{1},\;f_{2},\;f_{3},\;\ldots ,f_{2m}\right)}^{\prime }$ for each segment as follows

(13) $$f_{j}\left(var\right)=log\left[\frac{var\left[s_{j}\left(n\right)\right]}{\sum_{j=1}^{2m}var\left[s_{j}\left(n\right)\right]}\right]\;,\;j=1,\;2,\;\ldots ,\;2m$$

In the present study, the use of several additional metrics, namely, band power, energy, and entropy are investigated. The features based on band power and energy are given by

Band power (*BP*) [40]
  (14) $$\;f_{j}\left(BP\right)=log\left[\frac{1}{N}\sum_{n=1}^{N}{\left|s_{j}\left(n\right)\right|}^{2}\right]$$
Energy (*Eng*) [40]

(15) $$\;f_{j}\left(Eng\right)=\sum_{n=1}^{N}{\left|s_{j}\left(n\right)\right|}^{2}$$

Entropy is a metric that is commonly used for evaluating the complexity, regularity, and statistical quantification of time series. Multiple studies have shown that entropy may be used to analyze and establish biomarkers for a number of diseases, including epilepsy [41], attention deficit hyperactivity disorder [42], and autism [43]. This motivates us to look at using entropy as a method for identifying Parkinson’s disease. In the present study, instead of computing entropy directly from EEG data, it is proposed to compute it from the spatially filtered segment $S^{2m\times N}$, which may aid in the development of appropriate biomarkers for PD identification.

Several types of entropy are investigated in this work: Shannon entropy, norm entropy, threshold entropy, sure entropy, and log energy entropy. These metrics are defined as follows. If *k* is the number of unique values in the discrete signal $s_{j}\left(n\right)$ and $x_{i}$ is the probability frequency of the *i*th unique value, then the entropy features $f_{j}$ are given by:

Threshold entropy (ThEn) [44]
  (16) $$\;f_{j}\left(\mathrm{ThEn}\right)=\#\left\{i\;\mathrm{such}\;\mathrm{that}\;\left|x_{i}\right|>\alpha \right\}\;$$
  where ThEn is the number of time instants for which the signal is greater than a threshold α. The threshold is set to 0.2 based on trial-and-error to obtain the best accuracy.
Norm entropy (NoEn) [44]
  (17) $$f_{j}\left(\mathrm{NoEn}\right)=\sum_{i=1}^{k}{\left|x_{i}\right|}^{p}\;$$
  where p is the power of the entropy and must be such that 1 ≤ p. In this study, it is selected to be 1.1.
Sure entropy (SuEn) [44]
  (18) $$\;f_{j}\left(\mathrm{SuEn}\right)=k-\#\left\{i\;\mathrm{such}\;\mathrm{that}\;\left|x_{i}\right|\leq q\right\}+\sum_{i}\min\left(x_{i}^{2},\;q^{2}\right)\;$$
  where q is the threshold value, and usually >2. In the present study, it is selected to be 3.
Log energy entropy (LogEn) [44]
  (19) $$\;f_{j}\left(\mathrm{LogEn}\right)=\sum_{i=1}^{k}log{\left|x_{i}\right|}^{2}\;$$
Shannon entropy (ShEn) [44]
  (20) $$\;f_{j}\left(\mathrm{ShEn}\right)=\sum_{i=1}^{k}{\left|x_{i}\right|}^{2}log{\left|x_{i}\right|}^{2}\;$$

A feature vector f of length d is extracted from each EEG segment by filtering it using CSP and then computing one of the above metrics. The size of the resulting feature matrix is M×d, where M denotes the number of segments and d denotes the reduction number. Later on, the effects of M and d on the classification accuracy of PD vs. HC are investigated. In the following subsection, the classification methods and cross-validation stages are described.

### 2.4. Classification and Problem Formulations

In this study, a number of commonly used classification approaches to distinguish between PD and HC features are applied: bagging-based RF, LDA, QDA, quadratic kernel-based SVM, and KNN (kn = 3). The goal is to compare them and see which one produces the best outcomes in terms of off–PD/on–PD against HC classification. A detailed description of these classification methods can be found in [45,46,47,48].

The primary goal of this research is to detect Parkinson’s disease in individuals who are in an off-medication state and to distinguish them from those in the healthy control group. Due to the variety of data sets and conditions under which they were obtained, several classification problem formulations are considered and summarized in Table 3.

### 2.5. Performance Evaluation

In this study, several methods to evaluate the performance of the developed classification models are used: classification accuracy, sensitivity, specificity, F-score, and receiver operating characteristic (ROC) curve. The classification accuracy (CA) is given by

(21) $$CA=\frac{N_{correct}}{N_{total}}\times 100\%\;$$

where *N_total_* denotes the total number of feature vectors to be classified, and *N_correct_* denotes the number of feature vectors that are correct. For binary classification, the following formula can also be used to calculate accuracy in terms of the number of positive and negative predictions:

(22) $$CA=\frac{TP+TN}{TP+TN+FP+FN}\times 100\%$$

where *TP* = #True Positives, *FP* = #False Positives, *TN* = #True Negatives, and *FN* = #False Negatives. The sensitivity also called *recall* or *true positive rate* (TPR), indicates the ability of a classification model to correctly identify patients with the disease. On the other hand, the specificity, also called the *true negative rate* (TNR), indicates the ability of a classification model to correctly identify people without the disease. The sensitivity and specificity are defined by [49]:

(23) $$Sensitivity=\frac{TP}{TP+FN}\times 100\%$$

(24) $$Specificity=\frac{TN}{TN+FP}\times 100\%$$

The Precision metric quantifies the number of correct positive predictions made. In this study, the *F-score* is adopted since it provides a way to combine both precision and sensitivity into a single measure. *F-score* is defined as follows

(25) $$F\text{-}score=2\times \frac{Precision\times Sensitivity}{Precision+Sensitivity}\times 100\%$$

where Precision is calculated as

(26) $$Precision=\frac{TP}{TP+FP}\times 100$$

In addition to the above performance metrics, the ROC curve is also evaluated. The ROC curve is a graphical illustration of how a test’s TPR (sensitivity) and FPR (1-specificity) differ from one another. The AUC (area under the ROC curve) is a commonly used metric for assessing the detection performance. Good classifiers are characterized by AUC values that are close to 1. More information on ROC-AUC curves can be found in [50].

A k-fold cross-validation technique is implemented to obtain a reliable performance evaluation for the proposed classification models. k=10 is utilized in all of our experiments, which divides the dataset into ten equal subsets, one for validation (test) and the other nine for training [51]. The technique of cross-validation is repeated ten times (10-fold) by changing the test and training subsets. Equations (21)–(26) are used to evaluate the classification performance at each round. Each performance measure is averaged over the ten rounds to produce a single classification measure.

**Figure 4:**
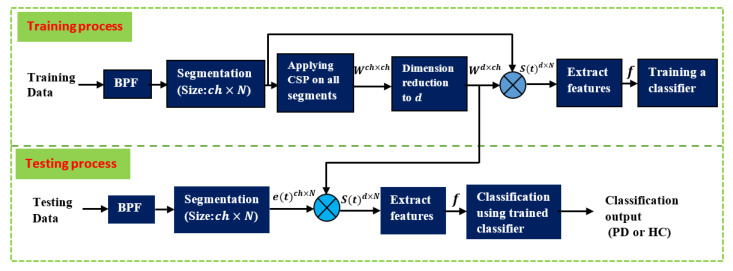
Summary of the processing and classification stages for the training and testing phases.

Figure 4 depicts the stages in which Parkinson’s patients’ and healthy people’s EEGs are processed during the training and test phases. As previously discussed, the data are initially separated into two parts: 90% of the data for training and 10% for testing. The training phase starts with BPF filtering of the training data. The filtered signals are then split into M equal segments, each with a size of ch×N. The number of segments is proportional to the length of each segment: the longer the segment, the lower the M, and vice versa. After dividing the signals, CSP is performed to all of the segments (including PD and HC) acquired using Equation (1) through Equation (10) to produce the projection matrix $W^{ch\times ch}$, which contains a set of CSP filters. The dimensionality of this matrix is then reduced by picking the first and last m filters to obtain the projection matrix $W^{d\times ch}$, as described in Equation (11). $W^{d\times ch}$ is then used to filter (multiply) each segment, as described in Equation (12). As a result, the size of each filtered segment is d×N. The next step is to create one feature vector f from each filtered segment, where the number of feature vectors is equal to the number of segments M. The number of elements in each feature vector is d: $f=\left(f_{1},\;f_{2},\;f_{3},\;\ldots ,f_{d}\right){}^{\prime }$. The elements of this vector are calculated using variance, energy, BP, or entropy according to Equations (13)–(20). The final step in the training phase is to train a classifier (RF, LDA, QDA, SVM, or KNN) using the feature vectors derived from the previous step. This concludes the training phase. In the testing phase, the test data subset is filtered with the same BPF and segmented in the same way as the training data. The difference here is that the CSP filters and its projection matrix are not recomputed. Instead, the same $W^{d\times ch}$ matrix created during the training phase is reused in the testing phase. The feature vectors are then created in a similar fashion to the training phase. The final step in the testing phase is to classify the test feature vectors using the classifiers that have been trained in the training phase to predict whether it belongs to PD or HC. The classification performance is then computed using Equations (21)–(26), with the cross-validation technique.

## 3. Results and Discussion

To verify the methods proposed here, two datasets from two different sources are used: SanDiego and UNM datasets. Because the datasets contain different states and conditions, as well as the diversity of the proposed methods, the results are presented in two separate subsections: SanDiego dataset-based results and UNM dataset-based results.

### 3.1. SanDiego Dataset Results

With this dataset, three classification problems are addressed: off-medication patients versus the healthy control group, on-medication patients versus the healthy control group, and off-medication patients versus on-medication patients, when the eyes are open.

#### 3.1.1. Off–Medication PD vs. Healthy Control

As the main problem for PD testing, the classification results of off–medication PD patients against a healthy control group are provided and discussed in this part. Each channel’s signal is fed into a 0.5–32 Hz BPF before being split into 606 non-overlapping 10-s segments (M=606). A total of 300 segments are acquired from PD patients, while 306 segments are obtained from HC patients. Each segment is transformed into a feature vector of length 32 (*d* = 32) using the proposed FE methods. This results in a feature matrix with a length of 606 × 32 that is then sent to the KNN classifier. The eight FE methods are listed in Table 4 and Table 5 together with their corresponding classification accuracy, sensitivity, specificity, and F-score results. Table 5 presents the findings for features extracted from CSP filtered signals, whereas Table 4 shows the results when CSP is not applied. For each feature extraction method, ten outcome values (classification accuracy, sensitivity, specificity, and *F*-value) are generated using 10-fold cross-validation. For each method, the average performance of the ten values is calculated, along with their standard deviation (mean ± st). The results show a great improvement when CSP is applied. When adopting CSP, the average classification accuracy of the ShEn technique, for example, rises from 75.27% to 91.91%. The CSP+Var and CSP+LogEn methods produce the best performance, with average classification accuracies of 96.37% and 94.22%, respectively. Other methods, such as CSP+Eng, CSP+LBP, and CSP+NoEn, have a classification accuracy of above 93%. In comparison to Table 4, the standard deviation values in Table 5 have been reduced. Table 5 shows that the CSP+ThEn and CSP+SuEn feature extraction methods have the worst performance. As a result, neither of these methods will be further investigated.

**Figure 5:**
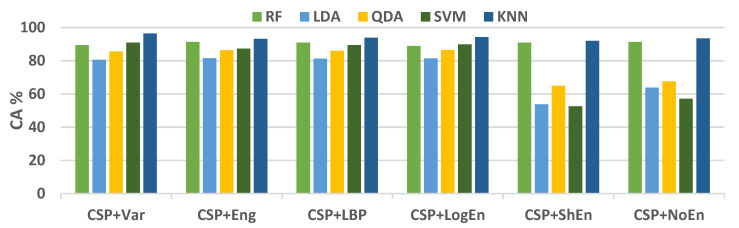
Average classification accuracy (off–PD vs. HC) using FR, LDA, QDA, SVM, and KNN.

**Figure 6:**
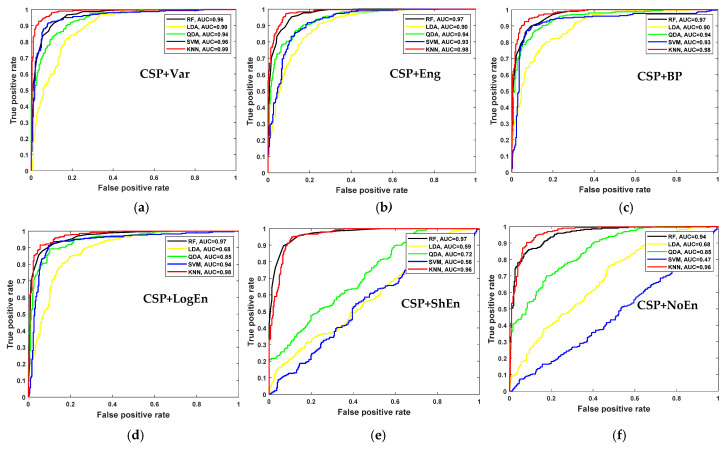
ROC and AUC of off–PD vs. HC classification based on features extracted from (**a**) CSP+Var, (**b**) CSP+Eng, (**c**) CSP+BP, (**d**) CSP+LogEn, (**e**) CSP+ShEn and (**f**) CSP+NoEn.

For further examination, four more classification algorithms are used in addition to KNN. Figure 5 compares the classification accuracy of RF, LDA, QDA, and SVM techniques applied to features extracted using all of the proposed methods. With all the FE methods, the KNN and RF classifiers achieve the highest classification accuracy and lowest standard deviation. Figure 6 presents ROC curves and AUC for the five classifiers. The KNN and RF classifiers have the highest AUC for all FE methods, whereas the LDA, QDA, and SVM have the lowest AUC. These results indicate that KNN consistently outperforms the other classifiers in terms of AUC, ROC, and accuracy.

##### Investigation of Frequency Bands

**Figure 7:**
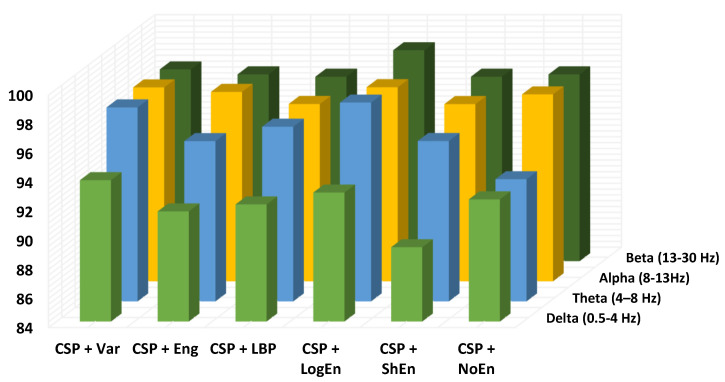
Classification accuracy of different EEG frequency bands using KNN (off–PD vs. HC).

EEG signals have a frequency range of 0 to 100 Hz, which is typically decomposed into five sub-bands: delta (<4 Hz), theta (4–8 Hz), alpha (8–13 Hz), beta (13–30 Hz), and gamma (>30 Hz). In this subsection, the aim is to find the sub-bands that lead to the highest PD classification performance. The gamma range has been eliminated from this study due to artifacts that may adversely affect the classification accuracy. Figure 7 depicts the classification accuracy when applying all of the proposed methods to different EEG sub-bands. According to the results shown in the figure (dark blue and yellow), in the highest subsection, our accuracies are acquired from beta and alpha. Because important information may not be concentrated in a single sub-band, the effect of combining two or more sub-bands on the classification performance is also being investigated. It can be seen from Table 6 that the frequency bands formed from both alpha and beta sub-bands lead to the highest classification accuracy. It is important to mention here that the CSP+LogEn method leads to higher classification accuracies compared to other FE methods. According to the results, the highest accuracy is obtained when the EEG signals are filtered using a 10–30-Hz band-pass filter.

##### Investigation of Reduction Number

**Figure 8:**
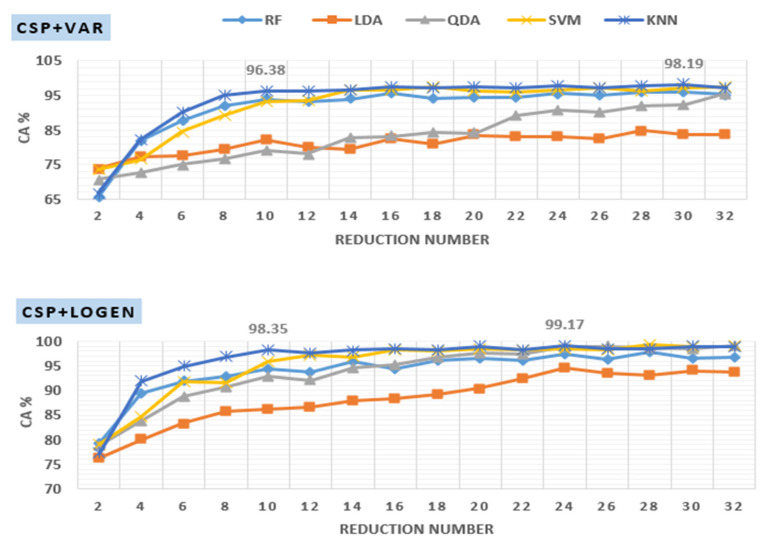
Effect of reduction number on KNN classification accuracy applied to features extracted using CSP+Var (**top**) and CSP+LogEn (**bottom**).

As previously discussed, the dimensionality of the ch×ch CSP projection matrix is reduced by picking only the first *m* and last *m* CSP filters resulting in a matrix $W_{CSP}$ with reduced dimension d×ch, where the reduction number d is equal to d=2m. Complexity can be minimized by reducing this number since the size of feature vectors is equal to d. On the other hand, choosing a very small d may lead to poor classification performance. Figure 8 presents the classification accuracies of off–PD versus HC signals filtered at 8–30 Hz with various d values. It is clear from the figure that the results are influenced greatly by the choice of *d*. In the case of the CSP+Var+KNN method, as the value of d increases from 2 to 10, the classification accuracy curve starts to significantly increase from 66% to 96.38% and then stabilizes or slightly increases after that. Similarly, in the case of CSP+LogEn+KNN, the classification accuracy curve begins to increase significantly from 77% to 98.35% at d=10 and then stabilizes or increases slightly thereafter. The optimal value of d, which leads to the highest classification accuracy, depends on several factors such as frequency band, FE method, classifier type, and others. In Figure 8, the highest classification accuracies for CSP+Var and CSP+LogEn are 98.19% and 99.17% obtained at d=30 and d=24, respectively, with the KNN classifier.

##### Investigation of Segment Length (SL)

Thus far, all of the signals have been split into 10-s segments. In this section, the effect of the segment length on the classification results is investigated. Because the highest classification accuracy is obtained by the CSP+LogEn FE method, it is used in this investigation. The BPF is also set to 10–30 Hz throughout all experiments. Table 7 presents the effect of the segment length along with the reduction number on the KNN classification performance. The table contains 72 outcomes, where the segment length is increased from 2 to 12 s while d is increased from 10 to 32. Decreasing the segment length leads to an increase in the number of segments M resulting in a larger number of feature vectors that are introduced to the classifier for training and validation. For example, the number of segments M equals 3032 when the segment length is 2 s while M decreases to 505 when the segment length increases to 12 s. According to the results in Table 7, there is a small improvement in the classification accuracy with decreasing segment length, especially at higher values of d. For example, at d=32, average classification accuracy is increased from 98.42% to 99.41% when segment length decreased from 12 to 2 s. At a segment length of 2 s and d=30, the highest classification accuracy of 99.47% is obtained by a combination of CSP+LogEn+KNN. At a segment length of 2 s and d=32, Table 8 shows the classification performance of RF, QDA, SVM, and KNN. The table demonstrates that the KNN classifier still outperforms the other classifiers, in terms of classification accuracy, sensitivity, specificity, and F-score.

#### 3.1.2. On–Medication PD vs. Health Control

This subsection presents and discusses the classification performance results of the on-medication patients vs. the healthy control group. For the purpose of consistency and to facilitate comparisons with the results of Section 3.1.1 (off–medication PD vs. health control), the reduction number is set to 32, frequency band to alpha and beta, and segment length to 10 s. The number of segments retrieved from on-medication patients is 270, whereas the healthy control group has 306 segments. As a result, there are a total of 603 feature vectors. Table 9 shows the same eight feature extraction methods that were used in Table 5 and their classification accuracy, sensitivity, specificity, and F-score as obtained with the KNN classifier. Similarly, the average performance and standard deviation are presented for each method. Table 9 shows that CSP+Var and CSP+LogEn FE methods achieve the best performance with an average classification of 92.87% and 92.85%, respectively. These two methods are the most effective for distinguishing between EEGs of on–PD patients and EEGs of the healthy control group. The results of these FE methods are bolded in Table 9. Similar to off–PD vs. HC classification, CSP+ThEn and CSP+SuEn FE methods provide low classification accuracy and high standard deviation. Consequently, these two methods are also excluded from further investigation.

**Figure 9:**
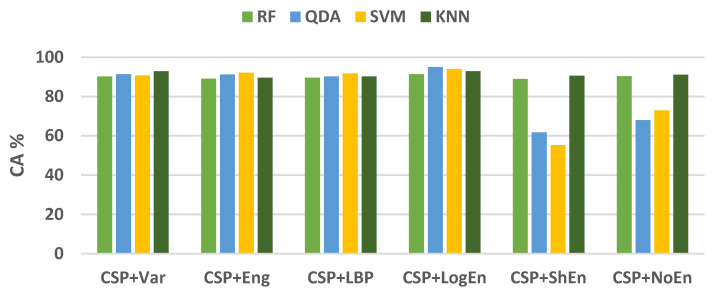
Average classification accuracy (on–PD vs. HC) using LDA, SVM, KNN, and LR.

**Figure 10:**
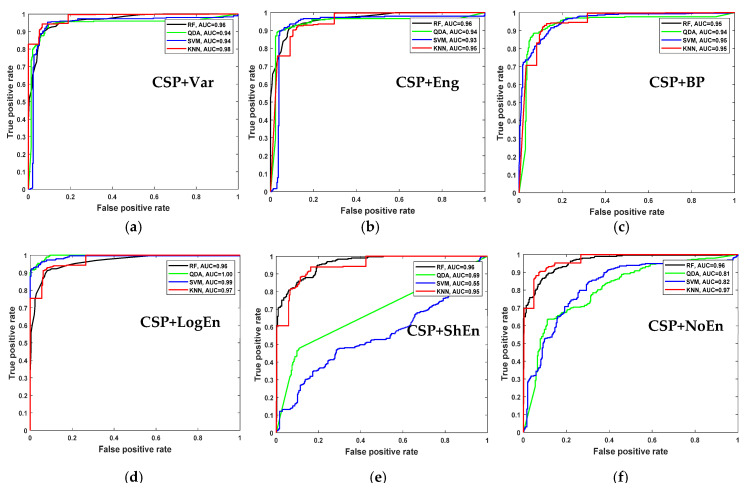
ROC and AUC of on–PD vs. HC classification based on features extracted from (**a**) CSP+Var, (**b**) CSP+Eng, (**c**) CSP+BP, (**d**) CSP+LogEn, (**e**) CSP+ShEn and (**f**) CSP+NoEn.

Figure 9 shows the average classification accuracy using RF, QDA, SVM, and KNN. Unlike Figure 5, there is no specific classifier that outperforms other classifiers in performance across all FE methods. For example, KNN outperforms other classifiers with the CSP+Var FE method, QDA is the best when coupled with the CSP+LogEn method, and SVM outperforms the rest with the CSP+Eng/LBP method. Figure 10 shows ROC curves along with AUC for the four classifiers. Except for CSP+LogEn, the KNN and RF classifiers deliver the highest AUC over all FE methods. In the case of CSP+LogEn, SVM and QDA achieve the highest AUC.

**Figure 11:**
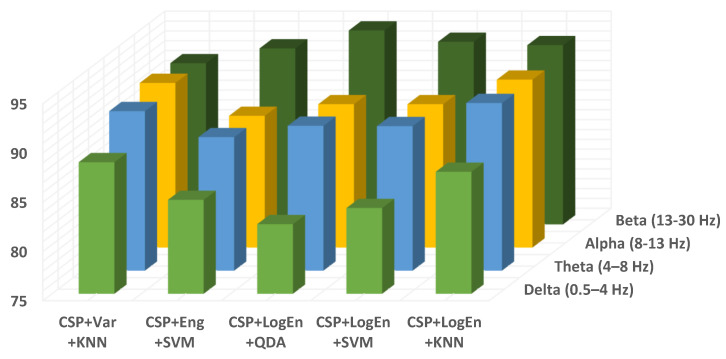
Classification accuracy of different EEG frequency bands using KNN of on–PD vs. HC.

In terms of the frequency band, Figure 11 shows the classification accuracy for all proposed methods when applied to different EEG sub-bands. Similar to off–PD vs. HC classification, the features extracted from the beta band are classified more precisely than others. Alpha and theta bands come in the second rank. Features extracted from the delta band lead to the worst classification accuracy. As result, the delta band is excluded from further investigation. Table 10 shows the KNN classification performance of features extracted from several frequency bands. It can also be noted from the table that the presence of the beta band within any wider frequency band improves the classification performance. For example, the features extracted from the 4–30 Hz frequency band are more accurately classified than those extracted from 4–13 Hz. Over all frequency bands, it can be seen that QDA and SVM classifiers deliver higher classification accuracy than KNN, especially with the CSP+LogEn FE method.

Next, the effect of changing the segment length for the on–PD vs. HC classification problem is considered. The BPF is set to 10–32 Hz for this investigation. Table 11 shows the classification results of features extracted by the CSP+LogEn method and classified by RF, QDA, SVM, and KNN. Results show that the effect of changing the segment length in this classification problem is small. For example, when decreasing the segment length from 12 s to 2 s, the accuracy changes from 91.46% to 92.25 and 93.24% to 93.38% for RF and KNN, respectively. At a segment length of 8 s, CSP+LogEn+SVM delivers the highest classification accuracy of 95.76%.

#### 3.1.3. Off–PD vs. On–PD

In this section, the classification performance results of off-medication versus on-medication patients are discussed. The purpose of this classification is to assess the effectiveness of the methods proposed in this study. Table 12 shows KNN classification performance with the following settings: 13–30 Hz, d=32, and a segment length of 2 s. The number of segments extracted is 2988 segments. It can be seen that, similar to off–PD vs. HC and on–PD vs. HC, the CSP+LogEn FE method outperforms other methods with an average classification accuracy of 97.52% and a standard deviation of 0.95.

### 3.2. UNM-Based Results

The proposed methods are also tested and validated using the UNM dataset in this section. Based on our findings using the SanDiego dataset, the frequency band is set to 10–30 Hz, the reduction number to 32 and the segment length to 2 s for all UNM-based experiments. CSP+Var, CSP+Eng, CSP+LBP, and CSP+LogEn FE methods are used with two states: open-eyes and close-eyes.

In the case of off–medication PD versus healthy control classification, the total number of the feature vectors is: 1620 (810 PD + 810 HC) for the open-eyes state and 1593 (810 PD + 783 HC) for the closed-eyes state. Table 13 includes the results of off–medication PD patients versus health control group classification using FR, SVM, and KNN classifiers. Two observations can be made from this table. The first is that the CSP+LogEn method achieves the highest classification accuracy, either in the case of open eyes or closed eyes, compared to the other FE methods. The highest classification accuracy of off–PD versus HC with the open-eyes state is 99.01%, which is obtained by the CSP+LogEn+KNN approach. In the case of the close-eyes state, 98.81% is the highest classification accuracy obtained by the same approach. The second observation is that there is not a big difference in the values of classification accuracy in both cases: the open-eyes and the closed-eyes states.

In the case of on–medication PD versus healthy control classification, the total number of the feature vectors is: 1650 (840 PD + 810 HC) for the open-eyes state and 1623 (840 PD + 783 HC) for the closed-eyes state. Table 14 includes the results of on–medication PD patients versus health control group classification. The highest classification accuracy of on-PD against HC with open eyes is 98.85%, obtained by both the CSP+Var+KNN and the CSP+LogEn+KNN approaches. In the case of the close-eyes state, the highest classification accuracy is 98.77% achieved by the CSP+LogEn+KNN approach. Like off–PD vs. HC, there is not a big difference in classification performance in both open-eyes and close-eyes states.

For further investigation and validation of the proposed methods, on–medication PD versus off–medication PD classification is also performed. The total number of feature vectors is 1650 (810 off–PD + 840 on–PD) for open eyes and 1593 (810 off–PD + 840 on–PD) for closed eyes. The results of this classification are presented in Table 15. As it can be seen from the table, our proposed methods achieve good performance.

Finally, the significance of this study can be evaluated by comparing the outcomes of the proposed methods to those of earlier studies. Table 16 compares our results to those of prior studies on Parkinson’s disease detection in the resting state. As seen in the table, the proposed methods in the present study achieve good performance using computationally efficient methods compared with other methods in the previous studies. The main advantages of our methods can be summarized as follows:

- The proposed methods are simple and computationally efficient, making their hardware implementation easier and faster in reality.
- The proposed methods are robust as they have been developed using a ten-fold CV.
- The proposed methods achieved good classification accuracy as it has been validated using two datasets from two different sources.
- To the best of our knowledge, we are the first group to present CSP-based methods for the detection of PD.

## 4. Limitations and Future Studies

Although the proposed methods are uncomplicated and perform well, there are some issues that need to be discussed.

- Channel selection: In the present study, all the signals coming from all channels are used, and CSP is applied to spatially filter the signals and reduce the number of features by decreasing the value of d. Selecting channels that contain only information important for the detection of Parkinson’s disease before applying signal processing was not exposed in this study. Future studies should be directed to using heuristic optimization methods to investigate the minimum number of channels that yield the maximum classification accuracy. PD detection using a few channels will be more practical and easier to use.
- Classification robustness: k-fold cross-validation is one of the most important techniques that are used to validate classification robustness. This technique was employed in all of the previous studies, shown in Table 16. In the present study, like in previous studies, k-fold cross-validation is also used to evaluate our proposed methods and compare their results with previous studies’ results. One of the disadvantages of this technique is that it may lead to the classification biasing problem resulting from data leakage. Therefore, future work includes the use of leave-one subject-out cross-validation along with k-fold.
- Source of data: One of the shortcomings of these types of studies is the use of different datasets, which makes the comparison of studies’ results unfair. It should specify a standard for evaluating the methods that are proposed by the researchers, including using public datasets. In the present study, two public datasets are used in order to compare the results of this study with those that used the same datasets. The authors also plan to test and confirm the proposed methods for additional brain disorders like autism and Alzheimer’s disease.

## 5. Conclusions

In recent years, EEG signal-analysis techniques have been used to diagnose brain abnormalities. This study focuses on the detection of Parkinson’s disease (PD) through the analysis and processing of EEG signals. Here, efficient common spatial pattern (CSP)-based methods for detecting Parkinson’s disease in two cases, namely, off/on–medication PD vs. healthy control group, are introduced. The extraction of the features from spatially filtered signals using different metrics, namely, band power, energy, and several types of entropies, is proposed, and the obtained results are compared with those of conventional CSP.

**Figure 12:**
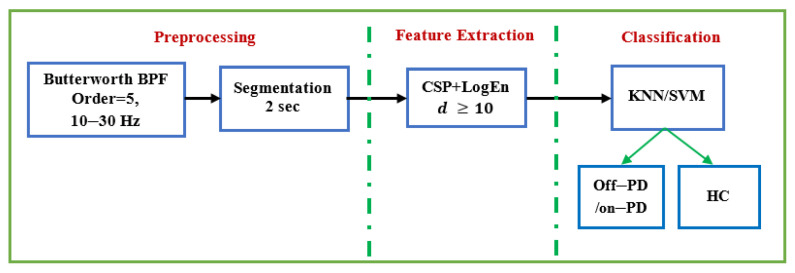
The complete method that provides the best performance.

This study also looks at how frequency bands and reduction numbers influence classification performance. Several classification algorithms are investigated to classify the extracted features. Two EEG datasets are used to evaluate the proposed methods: the SanDiego dataset (31 participants, 93 min) and the UNM dataset (54 subjects, 54 min). Results demonstrate that the combination of CSP and log energy entropy outperforms other FE methods, including conventional CSP. When compared to methods in the literature, the results show that the proposed method is able to achieve comparable classification performance. The results in terms of classification accuracy, sensitivity, specificity, and F-score for off–medication PD detection are 99.41%, 99.47%, 99.35%, and 99.40%, respectively. In the case of on–medication PD, performance results range from 95% to 98%. The findings also show that features extracted from the alpha and beta bands provide a higher classification accuracy. Figure 12 depicts a diagram of the entire procedure that produced the best results. These strategies produce outcomes that are encouraging and comparable to those found in earlier studies. In addition, our proposed method is completely portable and can be used in real-time PD diagnosis using EEG signals.

## Figures and Tables

**Table 1:** Summaries of methods of previous studies and their results.

Reference	FE Methods	Classifier(s)	Dataset	Classification Accuracy (%)
[29], 2018	Higher-order spectra (HOS)	DT, KNN, FKNN, NB, PNN, SVM	Malaysian dataset	90.6–99.6
[30], 2020	----	13 layer CNN	Malaysian dataset	88.25
[31], 2020	----	CNN+LSTM	UNM dataset	99.2
[32], 2020	PSD	Hyperplanes	UNM dataset	85.4
[33], 2021	--	CNN+RNN	Own dataset	99.2
[34], 2021	WT+statistical measures	SVM	SanDiego dataset	96.13

**Table 2:** Summary of information about datasets used in this study.

Dataset Name	PD Patients Information						HC Patients Information			
	Total No.	Age (mean ± st)	State(s)				Total No.	Age (mean ± st)	State(s)	
			Off	On	Open Eyes	Close Eyes			Open Eyes	Close Eyes
SanDiego	15	63.20 ± 8.20	Yes	Yes	Yes	No	16	63.50 ± 9.60	Yes	No
UNM	27	69.52 ± 8.66	Yes	Yes	Yes	Yes	27	69.52 ± 9.27	Yes	Yes

For SanDiego, the recording length for each person in each state is 3 min, while it is 1 min in UNM.

**Table 3:** Summary of the addressed classification problem formulations in the present study.

Classification Problem	Used Dataset	Problem Description
Open-eyes off–PD vs. HC	SanDiego and UNM	When the eyes are open, differentiate off–medication PD patients from the healthy control group
Open-eyes on–PD vs. HC	SanDiego and UNM	When the eyes are open, differentiate on–medication PD patients from the healthy control group.
Open-eyes off–PD vs. on-PD	SanDiego and UNM	When the eyes are open, differentiate off–medication PD patients from on–medication PD patients.
Close-eyes off–PD vs. HC	UNM	When the eyes are closed, differentiate off–medication PD patients from the healthy control group.
Close-eyes on–PD vs. HC	UNM	When the eyes are closed, differentiate on–medication PD patients from the healthy control group.
Close-eyes off–PD vs. on–PD	UNM	When the eyes are closed, differentiate off–medication PD patients from on–medication PD patients.

**Table 4:** Classification results of off–PD vs. HC using KNN classifier (without CSP).

FE Methods	Accuracy (%)	Sensitivity (%)	Specificity (%)	F-Score (%)
	mean ± st	mean ± st	mean ± st	mean ± st
Variance	78.21 ± 5.89	78.43 ± 5.81	78.41 ± 6.96	77.73 ± 6.41
Energy	86.63 ± 4.23	84.07 ± 4.66	90.14 ± 6.32	86.97 ± 4.22
LBP	86.64 ± 3.39	84.17 ± 4.81	89.97 ± 4.21	87.02 ± 3.19
LogEn	85.96 ± 4.69	83.78 ± 4.33	88.71 ± 6.47	86.22 ± 4.58
ShEn	75.27 ± 5.52	75.31 ± 6.61	75.58 ± 5.47	75.02 ± 5.38
ThEn	54.13 ± 8.82	53.38 ± 7.77	55.20 ± 10.45	58.54 ± 7.02
SuEn	79.36 ± 4.82	76.75 ± 4.76	82.93 ± 6.25	80.08 ± 4.73
NoEn	84.66 ± 1.89	83.56 ± 3.69	86.45 ± 4.12	84.76 ± 2.02

**Table 5:** Classification results of off–PD vs. HC using KNN classifier (with CSP).

FE Methods	Accuracy (%)	Sensitivity (%)	Specificity (%)	F-Score (%)
	mean ± st	mean ± st	mean ± st	mean ± st
CSP+Var	96.37 ± 3.18	96.80 ± 4.42	96.16 ± 3.21	96.34 ± 3.15
CSP+Eng	93.23 ± 2.53	91.21 ± 3.27	95.57 ± 2.74	93.34 ± 2.43
CSP+LBP	93.90 ± 2.19	92.11 ± 3.18	95.96 ± 2.65	93.97 ± 2.15
CSP+LogEn	94.22 ± 2.96	93.65 ± 4.11	95.27 ± 4.95	94.19 ± 3.01
CSP+ShEn	91.91 ± 4.72	92.40 ± 7.00	91.92 ± 3.95	91.90 ± 4.53
CSP+ThEn	49.67 ± 2.40	49.53 ± 1.37	59.81 ± 24.68	64.78 ± 2.42
CSP+SuEn	50.49 ± 5.45	50.17 ± 4.77	50.76 ± 6.59	53.55 ± 4.63
CSP+NoEn	93.39 ± 3.31	92.78 ± 5.25	94.45 ± 3.53	93.44 ± 3.18

**Table 6:** Effect of frequency bands on classification accuracy using KNN (off–PD vs. HC).

Frequency Band	FE Method					
	CSP+Var	CSP+Eng	CSP+LBP	CSP+LogEn	CSP+ShEn	CSP+NoEn
4–13 Hz	96.86 ± 2.55	96.52 ± 2.99	96.54 ± 3.13	98.18 ± 1.23	95.54 ± 2.10	98.18 ± 1.81
4–30 Hz	97.36 ± 2.09	96.20 ± 2.21	97.36 ± 2.10	98.02 ± 1.86	96.87 ± 1.79	97.86 ± 1.91
8–30 Hz	97.67 ± 1.58	97.04 ± 2.01	97.36 ± 1.77	98.85 ± 1.74	96.71 ± 1.54	97.20 ± 1.90
10–30 Hz	98.02 ± 1.51	98.02 ± 1.70	96.87 ± 2.12	99.34 ± 1.15	95.54 ± 3.22	97.17 ± 2.59
12–30 Hz	98.34 ± 1.57	97.85 ± 1.58	97.53 ± 3.03	99.17 ± 1.16	96.69 ± 2.47	98.18 ± 2.11
8–32 Hz	97.53 ± 1.39	96.54 ± 3.07	96.87 ± 2.50	98.68 ± 1.30	95.39 ± 2.65	97.68 ± 2.50
10–32 Hz	98.18 ± 1.65	97.52 ± 2.48	97.69 ± 1.60	98.85 ± 1.56	96.05 ± 2.59	98.69 ± 1.51
12–32 Hz	98.02 ± 1.30	97.36 ± 1.60	97.17 ± 2.58	98.36 ± 1.73	97.52 ± 1.94	98.52 ± 1.44
14–32 Hz	98.35 ± 1.09	97.52 ± 1.95	97.36 ± 1.96	98.84 ± 1.76	97.68 ± 2.50	98.35 ± 1.35
15–32 Hz	98.68 ± 1.05	96.86 ± 1.99	97.03 ± 1.87	98.18 ± 1.66	97.20 ± 2.33	97.03 ± 2.44
10–25 Hz	97.19 ± 1.77	96.70 ± 1.35	97.52 ± 2.11	98.18 ± 1.45	95.87 ± 2.85	97.69 ± 2.49
8–25 Hz	97.36 ± 1.77	97.02 ± 2.45	97.52 ± 1.61	98.52 ± 1.21	96.20 ± 2.61	97.19 ± 2.59

**Table 7:** The effect of segment length and reduction number on KNN classification accuracy based on CSP+LogEn FE method.

Reduction Number	Segment Length (Number of Segments *M*)					
	2 s (3032)	4 s (1516)	6 s (1010)	8 s (758)	10 s (606)	12 s (505)
10	98.02 ± 1.28	98.55 ± 0.87	97.62 ± 1.34	97.76 ± 1.53	98.02 ± 1.52	97.82 ± 2.37
12	98.71 ± 0.69	98.61 ± 0.79	98.91 ± 0.87	98.03 ± 1.27	98.84 ± 1.12	97.82 ± 2.57
14	98.88 ± 0.61	99.04 ± 0.54	98.81 ± 1.30	98.68 ± 1.38	99.01 ± 1.16	97.22 ± 2.14
16	98.98 ± 0.69	99.14 ± 0.88	98.51 ± 1.07	98.69 ± 1.07	98.35 ± 1.34	98.42 ± 1.80
18	99.04 ± 0.69	99.34 ± 0.70	98.91 ± 1.19	98.94 ± 1.05	98.84 ± 1.36	98.81 ± 1.03
20	98.94 ± 0.66	99.41 ± 0.85	98.71 ± 1.05	98.95 ± 0.83	99.01 ± 1.16	99.21 ± 1.37
22	99.18 ± 0.61	99.08 ± 0.83	98.71 ± 1.15	98.81 ± 1.31	98.84 ± 1.76	97.81 ± 2.19
24	99.14 ± 0.35	99.27 ± 0.66	98.91 ± 1.19	98.68 ± 1.25	98.84 ± 1.12	98.22 ± 1.46
26	99.08 ± 0.73	99.14 ± 0.77	98.61 ± 1.42	99.07 ± 0.89	98.85 ± 1.56	99.01 ± 1.04
28	99.41 ± 0.43	99.14 ± 0.54	98.51 ± 0.96	99.21 ± 0.93	98.68 ± 1.04	99.20 ± 1.03
30	99.47 ± 0.35	99.01 ± 0.47	98.91 ± 0.73	98.55 ± 1.16	98.19 ± 1.63	99.00 ± 1.41
32	99.41 ± 0.43	99.08 ± 0.77	98.22 ± 1.46	98.55 ± 1.58	99.17 ± 1.18	98.42 ± 2.03

**Table 8:** Classification results of off–PD vs. HC based on CSP+LogEn (10–30 Hz, SL = 2 s and *d* = 32).

Classifier	Accuracy (%)	Sensitivity (%)	Specificity (%)	F-Score (%)
	mean ± st	mean ± st	mean ± st	mean ± st
RF	97.59 ± 0.96	96.96 ± 1.99	98.30 ± 1.10	97.59 ± 0.94
QDA	98.58 ± 0.97	98.29 ± 1.50	98.88 ± 0.70	98.58 ± 0.97
SVM	99.04 ± 0.61	99.14 ± 1.03	98.97 ± 0.85	98.03 ± 0.62
KNN	**99.41** ± 0.43	**99.47** ± 0.80	**99.35** ± 0.74	**99.40** ± 0.44

**Table 9:** Classification results of on–PD vs. HC using KNN classifier.

FE Methods	Accuracy (%)	Sensitivity (%)	Specificity (%)	F-Score (%)
	mean ± st	mean ± st	mean ± st	mean ± st
CSP+Var	92.87 ± 2.32	92.02 ± 4.11	94.23 ± 4.55	92.83 ± 2.40
CSP+Eng	89.54 ± 4.24	87.47 ± 6.71	87.47 ± 6.71	92.45 ± 3.95
CSP+LBP	90.21 ± 1.85	89.88 ± 6.31	91.92 ± 5.41	90.16 ± 1.76
CSP+LogEn	92.85 ± 2.65	92.22 ± 5.72	94.33 ± 3.93	92.87 ± 2.48
CSP+ShEn	90.55 ± 3.43	88.95 ± 2.76	92.34 ± 4.98	90.54 ± 3.49
CSP+ThEn	51.58 ± 4.08	50.44 ± 2.31	61.83 ± 31.55	65.34 ± 3.47
CSP+SuEn	57.36 ± 5.36	56.66 ± 5.31	58.11 ± 5.47	58.12 ± 4.25
CSP+NoEn	91.03 ± 3.35	89.68 ± 4.01	92.88 ± 5.27	91.00 ± 3.54

**Table 10:** The effect of frequency bands on classification accuracy using KNN (on–PD vs. HC).

Frequency Band	Method				
	CSP+Var +KNN	CSP+Eng +SVM	CSP+LogEn +QDA	CSP+LogEn +SVM	CSP+LogEn +KNN
4–13 Hz	91.54 ± 3.08	91.04 ± 5.20	92.87 ± 3.69	93.20 ± 2.53	92.20 ± 2.95
4–30 Hz	92.37 ± 2.50	92.36 ± 3.16	95.20 ± 2.63	95.70 ± 1.94	93.20 ± 1.42
8–30 Hz	93.05 ± 2.53	91.56 ± 3.97	94.36 ± 1.94	94.70 ± 2.18	92.21 ± 2.93
10–30 Hz	92.87 ± 1.77	92.38 ± 2.81	94.53 ± 3.02	94.52 ± 2.49	92.21 ± 3.32
12–30 Hz	92.70 ± 2.75	92.53 ± 3.37	95.36 ± 2.90	94.53 ± 1.91	92.88 ± 2.89
8–32 Hz	91.55 ± 3.44	92.37 ± 4.32	94.54 ± 1.87	94.86 ± 1.97	92.88 ± 2.04
10–32 Hz	92.04 ± 3.40	92.37 ± 4.24	95.35 ± 2.92	94.20 ± 3.08	92.19 ± 3.64
12–32 Hz	92.20 ± 2.49	93.22 ± 4.32	95.20 ± 1.97	94.53 ± 2.93	93.54 ± 2.74
14–32 Hz	92.54 ± 3.41	92.54 ± 1.15	95.28 ± 2.97	94.37 ± 2.20	93.04 ± 2.79
15–30 Hz	91.39 ± 3.15	91.88 ± 3.99	95.18 ± 1.88	94.67 ± 2.76	93.85 ± 1.81
10–25 Hz	92.04 ± 1.90	93.87 ± 1.74	95.52 ± 2.36	94.52 ± 2.62	92.20 ± 2.72
8–25 Hz	92.05 ± 3.27	92.85 ± 1.63	95.35 ± 3.03	95.17 ± 2.18	92.51 ± 3.56

**Table 11:** The effect of segment length on the classification performance of on–PD vs. HC.

Classifier	Segment Length (Number of Segments)					
	2 s (3020)	4 s (1510)	6 s (1006)	8 s (755)	10 s (603)	12 s (503)
RF	92.25 ± 0.86	92.51 ± 2.46	92.14 ± 2.85	91.53 ± 3.99	91.53 ± 4.54	91.46 ± 5.14
QDA	93.54 ± 1.70	95.03 ± 1.72	94.32 ± 3.56	95.63 ± 2.99	95.19 ± 2.15	95.24 ± 3.12
SVM	95.00 ± 0.94	94.63 ± 2.64	94.32 ± 3.13	**95.76 ± 2.56**	94.53 ± 2.59	95.03 ± 2.14
KNN	93.38 ± 0.81	93.84 ± 2.25	93.43 ± 3.21	93.38 ± 3.35	92.87 ± 2.70	93.25 ± 3.41

**Table 12:** Classification results of off–PD vs. on–PD using KNN classifier.

FE Methods	Accuracy (%)	Sensitivity (%)	Specificity (%)	F-Score (%)
	mean ± st	mean ± st	mean ± st	mean ± st
CSP+Var	97.02 ± 1.06	97.59 ± 1.49	96.50 ± 1.50	97.02 ± 1.07
CSP+Eng	97.09 ± 0.79	97.46 ± 0.97	96.75 ± 1.19	97.09 ± 0.80
CSP+LBP	96.99 ± 0.44	96.95 ± 0.90	97.06 ± 1.15	97.00 ± 0.45
CSP+LogEn	**97.52 ± 0.95**	**97.92 ± 1.01**	**97.14 ± 1.22**	**97.52 ± 0.95**
CSP+ShEn	94.85 ± 1.28	96.32 ± 1.65	93.53 ± 2.16	94.78 ± 1.34
CSP+NoEn	96.15 ± 1.00	96.81 ± 2.04	95.58 ± 1.19	96.15 ± 0.96

**Table 13:** Classification results of off–PD vs. HC (UNM dataset).

	Classification Accuracy (mean ± st)					
	RF		SVM		KNN	
FE Methods	Close Eyes	Open Eyes	Close Eyes	Open Eyes	Close Eyes	Open Eyes
CSP+Var	96.23 ± 1.85	96.54 ± 1.02	97.17 ± 1.43	97.35 ± 0.92	98.24 ± 0.77	98.52 ± 0.88
CSP+Eng	96.99 ± 1.95	96.73 ± 0.72	97.05 ± 1.19	97.41 ± 2.05	98.12 ± 1.19	98.02 ± 1.04
CSP+LBP	96.61 ± 1.19	97.04 ± 1.53	96.92 ± 1.43	96.85 ± 1.68	98.05 ± 1.00	98.40 ± 0.83
CSP+LogEn	97.18 ± 1.19	98.02 ± 0.96	98.12 ± 1.11	98.58 ± 1.01	98.81 ± 1.00	**99.01 ± 0.93**

**Table 14:** Classification results of on–PD vs. HC (UNM dataset).

	Classification Accuracy (mean ± st)					
	RF		SVM		KNN	
FE Methods	Close Eyes	Open Eyes	Close Eyes	Open Eyes	Close Eyes	Open Eyes
CSP+Var	96.80 ± 1.45	96.91 ± 1.29	97.10 ± 1.27	98.12 ± 1.26	98.58 ± 0.87	98.85 ± 0.97
CSP+Eng	97.35 ± 1.54	97.58 ± 1.25	97.66 ± 1.59	97.76 ± 1.11	98.33 ± 1.13	98.18 ± 0.57
CSP+LBP	97.66 ± 1.19	97.45 ± 1.21	97.10 ± 0.97	97.58 ± 1.59	98.21 ± 0.79	98.42 ± 1.28
CSP+LogEn	97.29 ± 0.83	98.00 ± 0.57	97.54 ± 1.33	98.12 ± 0.73	98.77 ± 0.71	**98.85 ± 0.54**

**Table 15:** Classification results of off–PD vs. on–PD (UNM dataset).

	Classification Accuracy (mean ± st)					
	RF		SVM		KNN	
FE Methods	Close Eyes	Open Eyes	Close Eyes	Open Eyes	Close Eyes	Open Eyes
CSP+Var	95.82 ± 1.75	94.73 ± 1.57	97.88 ± 0.96	96.61 ± 1.28	98.36 ± 1.11	98.61 ± 0.50
CSP+Eng	96.67 ± 0.91	95.94 ± 1.40	97.52 ± 1.01	95.27 ± 1.61	98.79 ± 1.03	98.18 ± 0.70
CSP+LBP	97.27 ± 0.77	95.88 ± 1.14	97.88 ± 1.04	95.09 ± 2.70	98.79 ± 1.07	98.24 ± 0.60
CSP+LogEn	95.88 ± 1.53	94.61 ± 2.48	97.82 ± 1.04	96.73 ± 1.22	**98.97 ± 0.70**	98.73 ± 0.78

**Table 16:** Comparisons of our results with the results of previous studies (resting-state).

Reference	FE Methods	Classifier(s)	Dataset	Classification Type	Classification Accuracy (%)
Yuvaraj, R. et al. (2018)	Higher-order spectra (HOS)	DT, KNN, FKNN, NB, PNN, SVM	Malaysian dataset	Off–PD vs. HC	90.6–99.6
Oh, S. L. et al. (2020)	----	13 layer CNN	Malaysian dataset	Off–PD vs. HC	88.25
Shah S. A. et al. (2020)	----	CNN+LSTM	UNM dataset	Off–PD vs. On–PD	99.2
Fahim A. et al. (2020)	PSD	Hyperplanes	UNM dataset	Off–PD vs. HC	85.3
Lee S. et al. (2021)	--	CNN+RNN	Own dataset	Off–PD vs. HC	99.2
Smith K. K. et al. (2021)	WT+statistical measures	SVM	SanDiego dataset	Off–PD vs. HC On–PD vs. HC	96.13 97.65
Present study	CSP+LogEn	KNN	UNM dataset (Close/open)	Off–PD vs. on–PD On–PD vs. HC Off–PD vs. HC	98.73/98.97 98.77/98.85 98.81/99.01
	CSP+LogEn	KNN SVM KNN	SanDiego dataset	Off–PD vs. on–PD On–PD vs. HC Off–PD vs. HC	97.52 95.76 99.41

## Data Availability

Two publicly available datasets are used in this paper and they can be downloaded from: SanDiego dataset: https://openneuro.org/datasets/ds002778/versions/1.0.2. UNM dataset (d002): http://predict.cs.unm.edu/downloads.php.
